# SynPo-Net—Accurate and Fast CNN-Based 6DoF Object Pose Estimation Using Synthetic Training

**DOI:** 10.3390/s21010300

**Published:** 2021-01-05

**Authors:** Yongzhi Su, Jason Rambach, Alain Pagani, Didier Stricker

**Affiliations:** 1 TU Kaiserslautern, 67663 Kaiserslautern, Germany; Didier.Stricker@dfki.de; 2 German Research Center for Artificial Intelligence (DFKI), 67663 Kaiserslautern, Germany; alain.pagani@dfki.de

**Keywords:** object pose estimation, convolutional neural networks, training with synthetic images, deep learning, domain adaptation, 6DoF object pose, 6DoF object tracking

## Abstract

Estimation and tracking of 6DoF poses of objects in images is a challenging problem of great importance for robotic interaction and augmented reality. Recent approaches applying deep neural networks for pose estimation have shown encouraging results. However, most of them rely on training with real images of objects with severe limitations concerning ground truth pose acquisition, full coverage of possible poses, and training dataset scaling and generalization capability. This paper presents a novel approach using a Convolutional Neural Network (CNN) trained exclusively on single-channel Synthetic images of objects to regress 6DoF object Poses directly (**SynPo-Net**). The proposed SynPo-Net is a network architecture specifically designed for pose regression and a proposed domain adaptation scheme transforming real and synthetic images into an intermediate domain that is better fit for establishing correspondences. The extensive evaluation shows that our approach significantly outperforms the state-of-the-art using synthetic training in terms of both accuracy and speed. Our system can be used to estimate the 6DoF pose from a single frame, or be integrated into a tracking system to provide the initial pose.

## 1. Introduction

Robotic interaction plays an essential role in automatic production, showing a significant increase in demand in recent years [1]. At the same time, Augmented Reality (AR) has shown great potential in tasks such as maintenance and training [2,3], proving its ability to improve the efficiency of cognitive tasks. 6 Degree-of-Freedom (6DoF) pose estimation and tracking is a crucial technology for AR and robotic grasping tasks and has therefore recently received increasing attention by the computer vision and robotics communities.

Approaches relying on depth images exclusively or in conjunction with RGB images have achieved admirable results over the last years [4,5]. Depth information enables a more reliable pose estimation for low-textured objects, especially under challenging lighting conditions. However, depth information, which can be obtained from stereo cameras or other sensors such as Time-of-Flight (ToF) cameras, is still a privilege of a small group of devices with specific cost and performance limitations.

In contrast, monocular camera setups are low-cost and more compact. They are already available on most current mobile devices. Therefore, pose estimation algorithms relying only on RGB image data are of great importance while posing significant challenges as well. Classical approaches with RGB images [2,6] extract hand-crafted features from images and use them in a predefined matching procedure. However, the gradient required for feature extraction is sensitive to motion blur. Moreover, typical features used in image processing, such as ORB features [7], have limitations in scaling, rotation and illumination variations of the targets. They also require target objects with strong edge features.

Deep learning based approaches and especially Convolutional Neural Networks (CNNs) have shown excellent results on many computer vision tasks, such as object detection and classification [8,9,10], image segmentation [11] or optical flow [12]. The works of Kendall et al. [13,14] were the first attempts to use CNNs for regression of 6DoF poses for place recognition and direct relocalization. After that, several learning based approaches followed, achieving good results on the object pose estimation problem [15,16,17,18]. The use of 3D pose refinement methods such as Iterative Closest Point (ICP) [19] or 2D methods [20,21] to improve the initial estimate appears to be of crucial importance for the performance of these pose estimation methods.

The training dataset is a critical factor for the performance of deep learning based methods since a large amount of representative data is required. For tasks such as image classification or object detection, ground truth can be easily manually labeled. Obtaining ground truth data of object 6DoF poses is a more challenging task. It requires dedicated setups, such as a robotic arm or a tracker with additional markers [4,22]. Such approaches are time-consuming and can only cover a limited variation of the object poses, scene illumination and background. Apart from that, the use of real data can negatively impact the ability of trained networks to generalize well in new environments.

Due to these reasons, the use of synthetic images rendered using 3D models of objects is very promising. Training with synthetic images simplifies creating datasets with a large number of images while obtaining ground truth of the 6DoF object pose is given directly by the rendering system. However, new challenges arise from the use of synthetic data since trained models need to overcome the representation gap between real and synthetic data. Thus, a further step of domain adaptation is necessary. Therefore, the domain adaptation problem in deep learning is a highly active field of research.

In our previous work [18], we introduced a novel approach to overcome the representation gap between synthetic and real images. We suggested using the pencil filter as an image processing step. Both synthetic and real images are transferred to the pencil filter domain before the image processing. In the work presented here we improve several parts of our pipeline, from network architecture to rotation representation and synthetic training data preparation to achieve a significant increase in accuracy that surpasses the current state-of-the-art as shown in an extensive experimental evaluation on different datasets. In detail, we propose the following novel contributions extending our previous work:

- A CNN network architecture specifically designed for increasing accuracy in pose estimation regression through the replacement of pooling layers with convolutional layers.
- The use of lie algebra rather than quaternions for angle representation and regression.
- An ablation study that quantitatively shows the positive effect of all main points of our proposed approach.
- An overall approach that outperforms the state-of-the-art in 6DoF object pose estimation under similar conditions (i.e., no depth images in training, training exclusively on synthetic images) while being computationally very efficient due to the revised network architecture.

**Figure 1:**
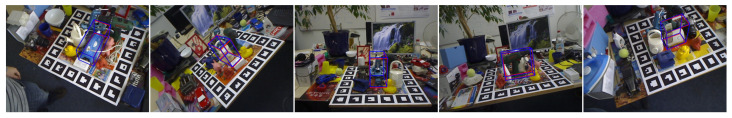
We visualize examples of the estimated pose using only SynPo-Net (without pose refinement). The groundtruth 3D bounding box and the predicted 3D bounding box are represented in red and blue, respectively.

Some examples of the estimated object pose can be seen in Figure 1. SynPo-Net can efficiently initialize a frame-to-frame tracking system like VisionLib [23] by providing an initial pose or relocalizing the system when tracking is lost.

The rest of this paper is organized as follows—in Section 2, we summarize existing work in object pose estimation and domain adaptation. In Section 3, we formulate the addressed problem of our work in detail. We introduce our approach in Section 4, discussing network architecture and training, dataset generation and domain adaptation. Subsequently, we present an extensive experimental evaluation of our approach and a comparison to the state-of-the-art in Section 5. Finally, we give concluding remarks in Section 6.

## 2. Related Work

In this section, previous work related to our approach is classified and summarized. We first give a short overview of object pose estimation methods using depth and color information (RGB-D). Subsequently, we discuss state-of-the-art methods relying only on RGB images, which is directly comparable to our work. Additionally, we look at existing synthetic to real domain adaptation techniques not limited to pose estimation problems but also problems of learning from images in general.

### 2.1. RGB-D Object Pose Estimation

In classical approaches, 2D and 3D features are extracted from the RGB-D source, and hypotheses are made and verified to match the object 3D model in the scene. In template-based approaches, for example, in the work of Hinterstoisser et al. [4], templates are generated in different viewpoints of the object model. The template consists of color gradient features in the object contour and the depth gradient features on the object surface. The combination of both intensity and depth information helps to provide a reliable matching result. ICP [19] and its variations are often applied to refine the estimated pose. The accuracy and speed of these template-based methods are heavily dependant on the number of used templates. In Reference [24], sub-linear matching complexity was achieved. However, this usually trades speed for accuracy. Tejani et al. [25] adapted Reference [4] into a scale-invariant framework to reduce the number of templates. Point pair features based approaches [5,26] match local features instead of the whole template of the object. In this way, local details which may be discriminating will not be ignored. However, such methods appear to be computationally more demanding.

With the development of deep learning, the features for template matching pipeline can be learned with CNNs. In Reference [27], a CNN was used to extract the descriptors of the object from various viewpoints. The approach of Reference [28] achieved significant improvement by using learned local RGB-D features rather than the gradient. Furthermore, Reference [29] proposed a CNN and a multi-view fusion framework to leverage the information from multiple images, which has advantages, especially in video datasets. Additionally, the use of CNNs enables per-pixel matching or per-pixel prediction. In Reference [30], the proposed framework fuses both pixel-wise features from the image and point-wise features from the corresponding depth image. Predictions are made with each of those fused dense features, and the highest confidence pose is chosen as the final prediction. The work of Reference [31] can be seen as an extension of Reference [32] in the RGB-D case. It addressed the pose estimation problem by keypoint voting in the depth map. The pose can be calculated by fitting the detected 2D keypoints to their corresponding 3D keypoints in the object model.

### 2.2. RGB Object Pose Estimation

Most object pose estimation approaches using only RGB images are realized through deep neural networks. The approaches can be broadly classified into three categories.

The first category is the approach of extending object detection algorithms. Based on the detected 2D bounding box, various methods can be used to estimate the object rotation. In SSD-6D [15], the rotation is treated as a discrete viewpoint classification problem. Other methods directly regress rotation in the form of quaternions [33] or a lie algebra [34] representation. To deal with the occlusion problem, Sundermeyer et al. [17] proposed an autoencoder-decoder structure to determine the rotation relying on the object representation in the neural network. However, as Su et al. in Reference [35] point out, the appearance of the object depends not only on the rotation but also on the translation. Estimating the object rotation without considering the bounding box position is not accurate. Reference [36] later solved this issue by introducing a perspective correction.

Another group of approaches regress the object pose from the entire RGB image directly. A first attempt to use a CNN for regression of 6DoF poses was PoseNet [13]. The GoogLeNet [37] architecture was used for camera relocalization from images showing moderate accuracy but the method was not evaluated for object pose estimation. Following the idea of using a holistic CNN solution for pose estimation, in Reference [18], a similar network was applied for the regression of object poses. The pencil filter was used as a domain adaptation technique to enable training exclusively with synthetic images.

Finally, the third category of approaches determines 3D/2D point correspondences and solves a Perspective n Point (PnP) problem. In contrast to appearance-feature based keypoints, CNNs can detect keypoints in a more complex feature space. For instance, in the work of References [16,38], the 2D projections of the 3D bounding box corners are detected. However, the corners of 3D bounding boxes are virtual keypoints that physically do not belong to the object. In Reference [32], a CNN was trained to predict vectors pointing to the keypoints pixel-wise. A robust RANSAC based voting scheme was used to locate the 2D keypoints using these vectors. More recently, dense per pixel 2D-3D correspondences could be obtained. Park et al. [39] used an autoencoder-decoder to generate object masks with color to obtain the dense 2D-3D correspondences, with the RGB value representing the predicted position in the model local coordinate.

### 2.3. Domain Adaptation Techniques

Several methods have been introduced to deal with the domain adaptation problem when learning from synthetic images. Different methods are often combined in practice to obtain improved results.

Creating synthetic images that resemble reality as much as possible (photo-realistic rendering) is probably the most obvious solution to the problem [40,41]. However, the material of objects and lighting in complex conditions are not easy to simulate. To increase the realism, rendering the object context-aware is also very popular [42]. For example, the object should be positioned on a table. This is required to detect all the plane and its rotation in the background image. Therefore, such approaches are computationally expensive while they tend to perform well only in controlled environments.

Domain adaptation with the help of real images from the target domain is also common. Some techniques perform some form of post-processing to the synthetic data to increase the similarity to real images. Learning approaches can be used with pairs of synthetic and real images [43], or Generative Adversarial Networks (GANs) [44] can be trained to generate realistic images from synthetic images [45,46]. Small amounts of real images can also be used to fine-tune the CNN [42].

The appearance of the RGB images can be affected a lot by the environment. So the domain adaptation for the RGB images is inherently not easy. In contrast, depth images provide only spatial information. The domain gap between real depth images and synthetic depth images is much smaller than for RGB images. The texture and illuminations have minimal effect on the depth images. Rad et al. [47] use the features from the depth images to predict the pose. This part can be trained using synthetic datasets, since the domain gap between depth images can be easily overcome. A mapping from the depth map features and color images features can be trained using real RGBD images obtained from depth sensors. Georgakis et al. [48] also learns the key points with depth images, and then learns to match the color image features to the key point features from the depth image.

Furthermore, it is common to perform random data augmentation as domain randomization, for example, random noise, random brightness and contrast, random backgrounds to object views and random textures on objects [15,49]. To further improve the diversity of data, Reference [50] also changed the shape of 3D models to get more training images. Trained with images from different domains, the CNN is forced to focus on the real critical part of the image, which is not randomized, that is, objects in our case.

Unlike other works that attempt to fit one domain into another, we use a different approach to solve the domain adaptation problem in this work. We transform both the real and synthetic images into a new domain where visual similarity is increased and adaptation is facilitated (details in Section 4.2). Our approach is a general approach, we do not require any images or prior knowledge from the target domain. Our domain adaptation method is used together with domain randomization for further improvement.

## 3. Problem Formulation

The 6DoF object pose can be described with a rotation and a translation from the object coordinate system *O* to the camera coordinate system *C*. The translation part can be expressed with a translation vector $O_{c}$$\in R^{3}$ representing the position of the object coordinate system origin in the camera coordinate system. The rotation can be formulated in many different ways. In this work, we use lie algebra $\phi_{\mathrm{co}}\in R^{3}$ with the footnote co denotes the rotation from object coordinate to camera coordinate.

In the frame of this work, we focus on the object pose estimation relying only on the color image, that is, given a single image, the pose of the target object should be estimated. Training of the proposed approaches is done exclusively with synthetic data.

## 4. Method

We describe the entire proposed pipeline of our object pose estimation system in this section. We first present the architecture of our SynPo-Net, which is the CNN designed for the task at hand, together with the used loss function in Section 4.1. Subsequently, we discuss how the predicted pose can be further refined, and the relationship between pose refinement and object 6DoF tracking in Section 4.1.3. In the end, we discuss the synthetic training data generation and the pencil filter as the proposed domain adaptation technique in Section 4.2).

### 4.1. 6DoF Object Pose Estimation

#### 4.1.1. SynPo-Net Architecture

Currently, most neural network structures are designed for image classification tasks. In this paper, we argue that when using such kinds of network structures for object pose estimation, certain modifications can improve performance. Specifically, we used the Inception network [37] as a base network and researched the impact of input resolution, the use of pooling layers, and the representation of rotation.

**Input Resolution**: Li et al. [51] first mentioned that it is not suitable to directly use the object classification CNN as the backbone for object detection. Object classification tasks only need to recognize the object class. For this purpose, a global overview of the image is of high interest, which can be achieved by applying a large downsampling factor. However, object detection methods still need to estimate the object position accurately. The spatial resolution has more meaning in this case. This argument also applies to object pose estimation tasks. In Reference [51], the downsampling factor is reduced to double the output spatial resolution of the last CNN layers. Rather than reducing the downsampling factor, we suggest increasing the resolution of the input image.

**Pooling Layers**: Max pooling layers are widely used in CNNs for object classification [37,52]. To recognize object classes regardless of the object position in the image, these CNNs are required to be less sensitive to the object position. The max pooling layer selects only the maximum value in the reception field of the kernel. In other words, if the input layer has been shifted within half of the kernel size, the output layer does not change. This might be beneficial for classification tasks but not for pose regression that needs to be sensitive even to small changes of the target position.

**Figure 2:**
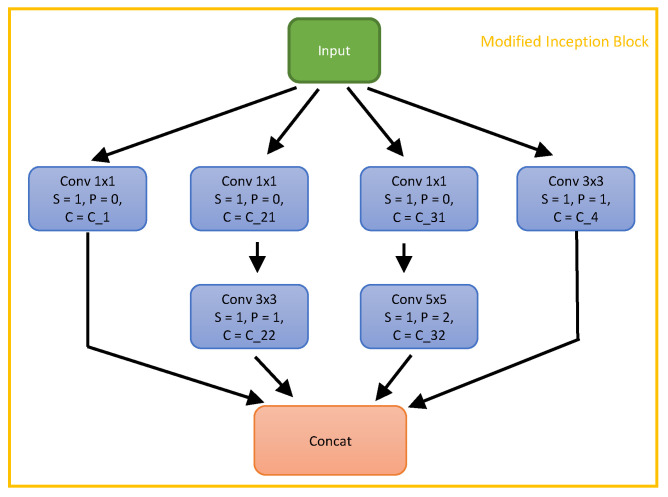
The proposed modified inception block. Each convolutional layer is followed by batch normalisation and a ReLU activation layer.

To avoid the use of max pooling layers, we replace them with convolutional layers. More specifically, for max pooling layers followed by a convolutional layer, we merge them to one convolutional layer, in which the kernel size and the stride are the same as the max pooling layer and the number of output channels is the same as the initial convolutional layer (see Figure 2 as an example). For max pooling layers followed by inception blocks, we replace the max pooling layer with a convolutional layer without changing the kernel size, the stride, and the input size so that the output channel size of the convolutional layer equals the max pooling layer input channel size. We also replace the average pooling layers with convolutional layers. The convolutional layers can be equivalent to average pooling layers when the weight is learned as $1/(kernel\_size^{2})$. So we suggest that this replacement can further increase the representative capacity of the network.

**Representation of Rotation**: Rotation matrices, Euler angles, quaternions and lie algebra are the most common representations of rotation. Rotation matrices can be used directly to rotate 3D points through matrix manipulation. However, using 9 elements to represent 3DoF transformations is unnecessarily redundant. Besides, rotation matrices need to be normalized, which introduces additional constraints to the optimization process.

Euler angles are easy to understand as a representation, and therefore commonly used for human-machine interaction. However, this representation is ambiguous, which means the same rotation can be represented with various combinations of Euler angles. Additionally, the gimbal lock problem creates essentially noncontinuous points during interpolation. These properties make Euler angles less suitable for optimization problems.

Quaternions are compact representations that consist of only 4 parameters and are unambiguous except that every quaternion is equal to the negative of itself. This representation also avoids the gimbal lock problem of Euler angles and allows a smooth interpolation for rotation [53]. Nevertheless, quaternions need to be normalized, which makes them suboptimal in regression tasks. (Details in Section 4.1.2).

Lie algebra so(3) is a representation of rotation extensively used in optimization problems. It is a compact 3-dimensional vector that can be mapped to a rotation matrix using the exponential map. At the same time, it is ambiguity free in an arbitrary 0∼2π interval and does not require additional constraints. Therefore, we propose using lie algebra as a representation of rotation for regression with a CNN.

**Other CNN Structure Adjustments**: To make sure the number of output channels of the convolutional layers increases smoothly, we added more layers. Additionally, the technique of batch normalisation [54] has been applied to accelerate the training process, which was not used in our previous work.

**Figure 3:**
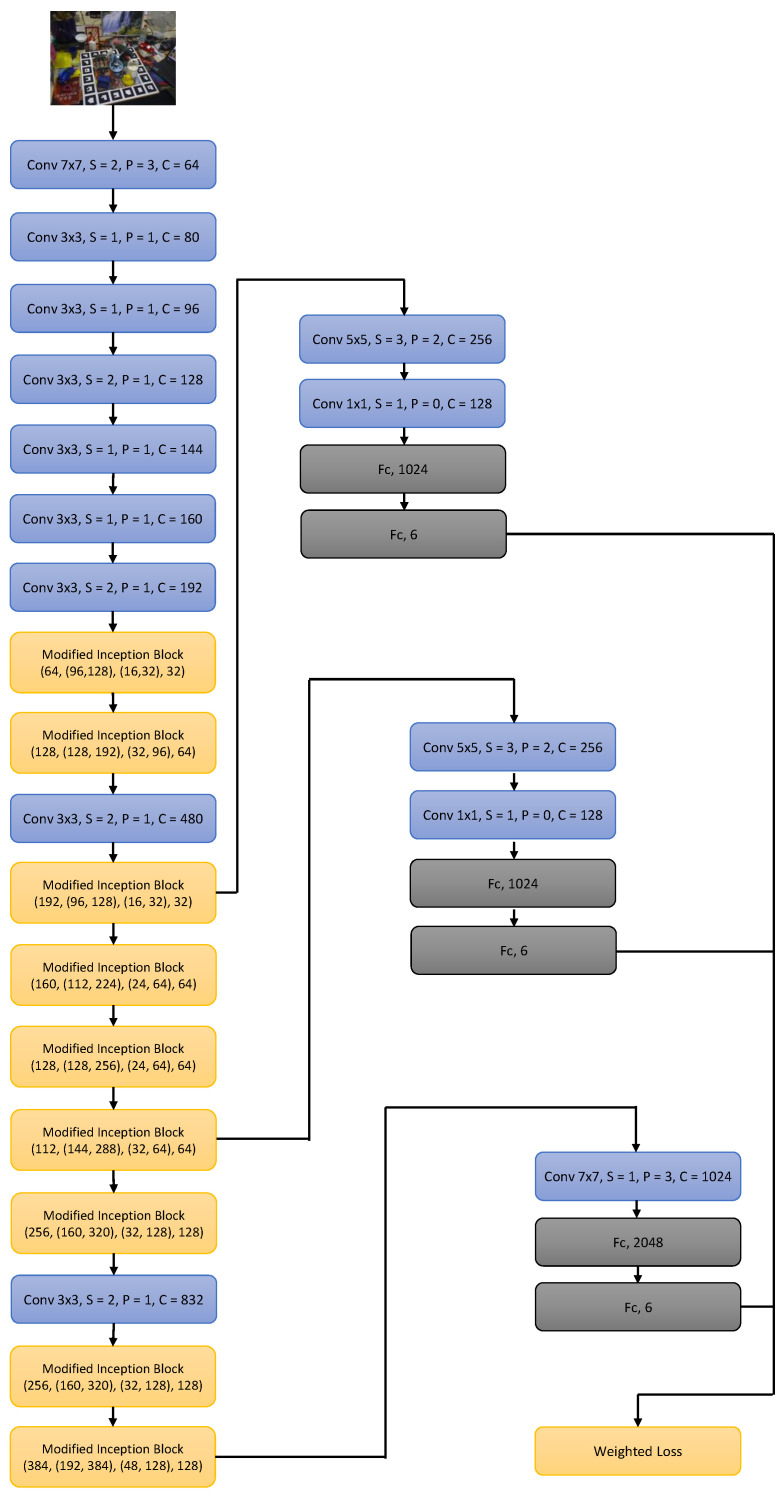
The proposed SynPo-Net architecture. Each convolutional layer is followed by batch normalization and a ReLU activation layer. The 6 (or 7) pose values regressed by the Convolutional Neural Network (CNN) represent the 3D translation and 3D rotation vector of lie algebra (or quaternion). The architecture variant with quaternions is only used for the ablation study.

Our proposed SynPo-Net is graphically represented in Figure 3.

#### 4.1.2. Loss Function Definition

We used L2-norm losses for both translation and rotation regression. In our previous work, we used quaternions to represent the rotation. In that case the loss function can be expressed as

(1) $$L_{\mathrm{balanced}\_O_{c}\_q}=∥O_{c}-\hat{O_{c}}{∥}_{2}+\alpha_{q}\cdot {∥q_{\mathrm{co}}-\hat{q_{\mathrm{co}}}∥}_{2}\;,$$

where $O_{c}$ and $q_{\mathrm{co}}$ are the predicted translation vector and rotation quaternion and $\hat{O_{c}}$ and $\hat{q_{\mathrm{co}}}$ are the respective ground truth values. Since the predicted quaternions are not restricted, we need to normalize them before they can be used to represent the rotation. $\alpha_{q}$ is the hyper-parameter used to balance the translation and rotation loss.

Using lie algebra to represent the rotation, the additional normalization can be avoided. Then the loss function can be formulated as

(2) $$L_{\mathrm{balanced}\_O_{c}\_\mathrm{lie}}=∥O_{c}-\hat{O_{c}}{∥}_{2}+\alpha_{l}\cdot {∥\phi_{\mathrm{co}}-\hat{\phi_{\mathrm{co}}}∥}_{2}\;,$$

with $\phi_{\mathrm{co}}$ represent the predicted lie algebra rotation and $\hat{\phi_{\mathrm{co}}}$ the ground truth rotation. Thus, the loss function with lie algebra is more straightforward for optimizing the object rotation (without the normalization step). We used the $L_{\mathrm{balanced}\_O_{c}\_\mathrm{lie}}$ to train SynPo-Net. Meanwhile, we also trained a CNN using the $L_{\mathrm{balanced}\_O_{c}\_q}$ only for comparison. The result can be found in the experimental section (see Section 5).

The loss functions discussed above is also calculated in the middle layers of the network as auxiliary losses and weighted into the primary loss of the network. Those auxiliary losses enable an effective gradient propagation in the lower layers and facilitate the training of the deep neural network. The weighted loss, which is used for the back propagation training, is defined as

(3) $$L_{\mathrm{weighted}}=\gamma_{1}\cdot L_{\mathrm{aux}\_1}+\gamma_{2}\cdot L_{\mathrm{aux}\_2}+\gamma_{3}\cdot L_{\mathrm{pri}},$$

$\gamma_{1}$, $\gamma_{2}$, $\gamma_{3}$ are the hyper-parameters to adjust the effect of the auxiliary and primary loss.

#### 4.1.3. Pose Refinement

Pose refinement is often used to improve the pose after an initial estimate is available, which can be optionally applied after the pose prediction from CNN. This task has a lot of similarities to frame-to-frame tracking. The tracking [21] or refinement [19,20] algorithm takes the information from previous frame or an initial pose and performs an improvement step of the estimate, often using geometry-based approaches. If the frame rate is high enough, we can expect that the pose difference between 2 continuous frames is minimal. In this case, the pose refinement algorithm can also be used for object tracking.

2D images are less sensitive to object depth translation than the object translation within the image plane. Thus, if depth is available, a pose refinement with depth information can help to estimate the pose very accurately. Since the LINEMOD dataset [4] also provides depth images. Similarly to other works, we also report the result with 3D refinement methods after using our proposed 2D-based estimation. We used the ICP algorithm for the pose refinement. Only the visible surface of the object is taken into account in each ICP iteration. The visible surface is updated after each iteration.

### 4.2. Training Dataset with Proposed Domain Adaptation Technique

A training dataset should cover as many as possible viewpoints of the object. Following the dataset generation pipeline described in Reference [18], we generated images of the objects from different viewpoints with random backgrounds from the PASCAL VOC [55] and the IKEA [56] datasets.

**Figure 4:**
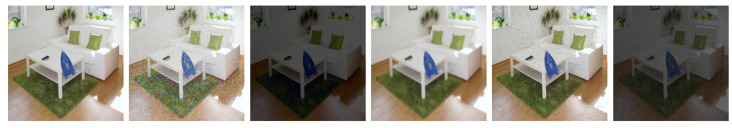
We apply randomly different kinds of effects on synthetic images before the application of the pencil filter. The examples of applied effects are shown from left to right: no effect, Gaussian noise, contrast and illumination changes, motion blur, speckle noise and mixture of all effects.

We augment our rendered training data by randomly adding various effects, that is, Gaussian noise, random contrast and brightness adjustment, motion blur, speckle noise (see Figure 4). In the previous work, the augmentations have been applied during the dataset generation. In this work, the augmentations are dynamically applied to the images every time they are loaded for training the network in a random way. Thus, the augmentations for a certain image are not fixed and the capacity of the dataset is increased further.

**Figure 5:**
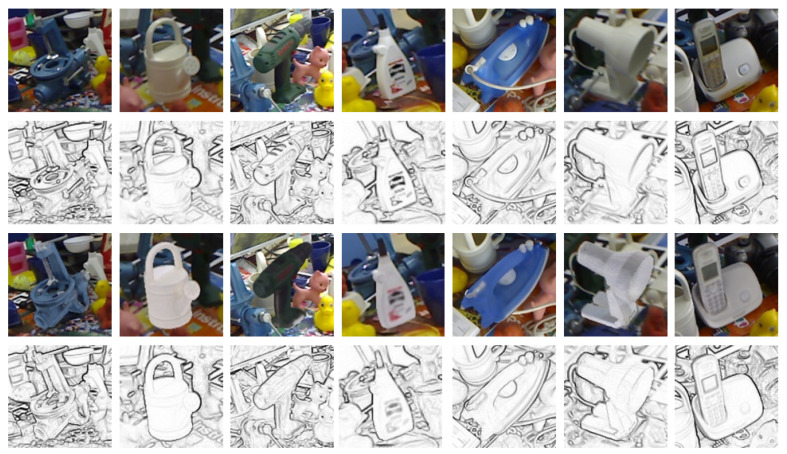
To visualize the effect of the pencil filter, we render the object model with the same pose over the real image. However, it should be noted that for training our network we only rendered the models on random backgrounds. First row: Cropped real images from the LINEMOD dataset [4]. Second row: Real images after applying pencil filter. Third row: Rendered images with same object pose and background as the first row. Fourth row: Rendered images after applying pencil filter.

Subsequently, the pencil filter is applied on the synthetic training dataset and the resulting images are then used to train the network following the method of Reference [18]. Unlike other domain adaptation techniques, which attempt to transform one domain to another, we transform both domains into a third intermediate domain, in which the similarity between synthetic and real images is increased. We avoid providing color information to the network, which can be volatile when applied across datasets with different illumination conditions or between synthetic and real images. Also, unlike the 3D reconstructed models, the colors of CAD models are usually different from those of the final products. We use images in the pencil filter domain where the more reliable edge information is enhanced. In Figure 5, we present several rendered and real images with their corresponding pencil filter version to show the increased similarity in the pencil filter domain. This abstraction of information, apart from being effective in domain adaptation, also allows us to decrease the input size to our network from an RGB image to a single-channel image, positively influencing training and forward pass time.

## 5. Evaluation

Our evaluation results are presented in this section. We have performed an ablation study with selected objects from the LINEMOD dataset [4] to investigate the effects of each proposed CNN design and training decision separately. Subsequently, we compare against the state-of-the-art by evaluating our proposed CNN on the entire LINEMOD and TUD-L [57] datasets. LINEMOD is the most commonly used benchmark for object pose estimation and TUD-L is a dataset focusing specifically on lighting variations.

### 5.1. Implementation Details

We implemented our CNN with MXNet [58] and trained the CNN on an Nvidia GeForce GTX 2080Ti GPU (Nvidia, Santa Clara, CA, USA). We used the same CNN settings for evaluating on both datasets. We set the hyper-parameters $\alpha_{q}$ and $\alpha_{l}$ both equal to 30 to balance the loss of translation and rotation, and {$\gamma_{1}$, $\gamma_{2}$, $\gamma_{3}$} as {10, 10, 40} to balance the effects of auxiliary losses and primary loss. We train our models using the ADAM optimizer [59] with a learning rate of 0.0002 and parameters $\beta_{1}=0.9$ and $\beta_{2}=0.99$. The models are trained for 800 epochs with a batch size of 16.

We created the training dataset with OpenGL. It consists of a number of 35,000–40,000 random poses per object. For the LINEMOD training dataset, we rendered the images without any light effect (the object’s color is provided directly from the 3D Model). For the TUD-L training dataset, 30% of the images are rendered without any light effect. For the rest 70% images, we applied only diffuse reflection (according to Phong lighting model [60]) with a random light source position (no specular reflection). The pencil filter was applied for the domain adaptation to the training and evaluation datasets.

### 5.2. Error Metrics

The Average Distinguishable Distance (ADD) error has been first proposed in Reference [4]. This error calculates the average distance of model points projected to the camera domain using the predicted pose to the same model points projected using the ground truth pose. ADD errors are compared to a threshold that is based on the object size (largest diameter) for fairness.

For better dealing with ambiguous cases including symmetry and occlusions, the Visible Surface Discrepancy (VSD) error was proposed in Reference [61] and optimized in Reference [57]. It measures distance difference in the depth image using only the visible part of the object in the image. The Maximum Symmetry-Aware Surface Distance (MSSD) error introduced in Reference [62] indicates the chance of successful grasp with the robot arm by focusing on the maximum prediction error rather than the average error in ADD. In contrast, maximum symmetry-aware projection distance (MSPD) is more suitable to evaluate the RGB-only methods that are used in the Benchmark for 6DoF Object Pose Estimation (BOP) [63]. The model points are projected into the image plane for the measurement, which overcomes the RGB-only method’s weakness. To take full advantage of the different metrics, a method’s average performance with the VSD, MSSD, and MSPD is used as the bop performance score. In our experiments, we use suitable metrics that allow comparison to the related state-of-the-art works. More specifically, we use ADD error for the evaluation of LINEMOD dataset and use bop performance score for the evaluation of TUD-L dataset.

### 5.3. Ablation Study

#### 5.3.1. The Pencil Filter Effect

In our previous work [18], we have already presented a qualitative proof that the accuracy can be improved by applying the pencil filter for domain adaptation. In this work, we try to intuitively represent the effect of pencil filter in reducing the difference between the real and synthetic images. We made the following experiment similar to in Reference [64].

We trained two networks separately with the same synthetic images of an object. One of them was trained with single-channel pencil images, and the other was trained with RGB images. Then we rendered the object above the real images with the corresponding ground truth pose. We test how the CNN is activated differently based on both types of images (the rendered images and the original real images). We passed both types of images to the network to observe the absolute differences in the feature map after the third modified inception block, based on which the first auxiliary pose is predicted.

**Figure 6:**
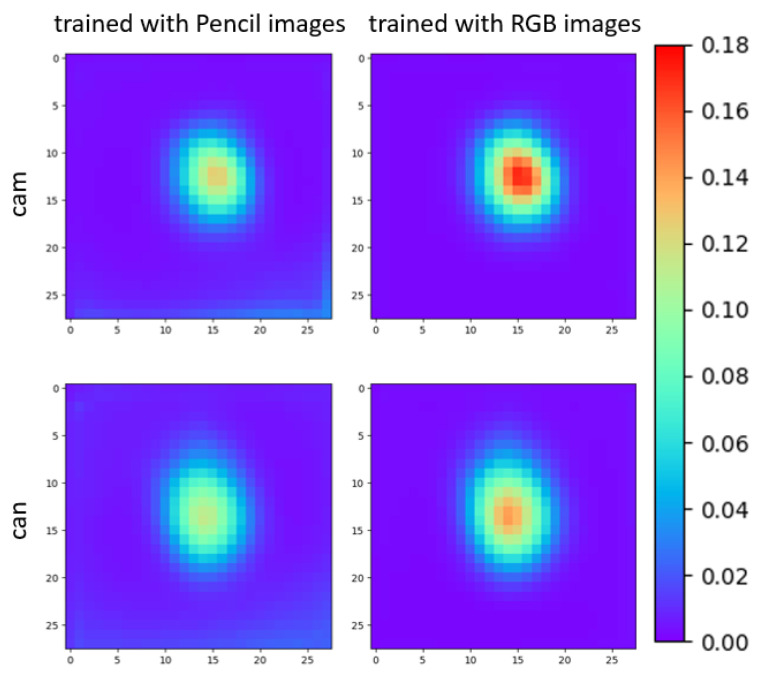
We compare the difference in activation of a CNN layer for a network trained with pencil images and with RGB images to illustrate the domain adaptation efficiency.

We first calculate the average absolute difference of this layer with all the images for the camera (cam) and watering can (can) in the LINEMOD dataset. The difference can be qualitatively represented with Figure 6. It is obvious that the maximum difference of the synthetic image and the real image is smaller in the pencil domain.

We also quantitatively report the {max absolute difference, mean absolute difference, standard deviation of absolute difference} in this averaged feature map of Figure 6. For the cam object trained with the pencil image, these values are {0.1254, 0.0126, 0.1146}, and trained with RGB images {0.1707, 0.0127, 0.1473} respectively. Despite the fact that pose estimation for the cam object has relatively low accuracy for our approach (see Section 5.4), the pencil filter still helps overcome the gap between the synthetic images and the real images. We achieved an outstanding result with the can object, and naturally, the absolute difference is even smaller than the case of the camera. For the network trained for the watering can, trained with pencil images, the values are {0.11309, 0.0159, 0.114}, and trained with RGB images {0.1414, 0.0137, 0.1398} respectively.

#### 5.3.2. CNN Architecture Modification Effects

Our main ablation study results are presented in Table 1. Here, we evaluate the influence on the pose estimation accuracy for each one of the proposed ideas. We use the driller object of the LINEMOD dataset as an example and evaluate all proposed CNN modifications in this paper. The pencil filter was applied in all experiments. In the first five experiments, batch normalization was not used, and we set their learning rate to 0.0001 and batch-size of 32 to make full use of the GPU Memory. The CNN in the second experiment corresponds to our previous work [18]. The result in the second experiment is slight better than we reported in Reference [18], because we adjusted the {$\gamma_{1}$, $\gamma_{2}$, $\gamma_{3}$} values. The last experiment with all the modifications corresponds to our proposed SynPo-Net, the training settings are described in Section 5.1.

According to the Table 1, we can see that all the proposed modifications in this paper truly help in improving the CNN’s performance for object pose estimation. Also, results indicate that the amount of data plays a crucial role. By applying random augmentations after the images have been loaded, we manage to increase the dataset diversity further and have a positive effect on the results.

### 5.4. LINEMOD Dataset State of the Art Comparison

We summarise the result of different methods on LINEMOD in Table 2 for comparison. The methods are divided into two groups based on the data type used for training. Our proposed CNN outperforms the state-of-the-art using synthetic training AAE [36] by a large margin for most objects and on average. However, their approach deals better with symmetric objects such as the glue and eggbox. Our approach removes color information and focuses more on edge information. The 3D model of the eggbox is coarse, which could also influence the quality of synthetic training datasets. Our CNN also performs weakly with the camera object. According to Figure 6, we can attribute the cause to the difference between the real image and synthetic image, as domain adaptation is not that successful in this case. Overall, our proposed method clearly shows the best-reported results thus far when training with synthetic data, and even exceeds Brachmann [65] and BB8 [16] which have been trained with real images. For further comparison, we also trained Pix2Pose [39] and YOLO6D [38] using the same synthetic images as ours (with all augmentations applied). For Pix2Pose [39], we provided the ground truth 2D detection bounding box. It is interesting to note that in contrast to the result when trained using real images, Pix2Pose [39] has a poor performance when trained using synthetic images. We think the dense-correspondence matching methods focus on the appearance of the object in pixel-level. Thus they are easier to overfit to the synthetic images and have problems generalizing to the real images, when trained solely using synthetic images.

In Table 3 we also report the results when ICP pose refinement is applied using depth images. Our result is better than that of Reference [36] after applying pose refinement as well. However, the projective ICP that was applied in Reference [15] takes leverage of both image and depth information and performs still best on average. This pose refinement approach is nevertheless not openly available for testing and experimentation. In any case, our approach still outperforms SSD-6D [15] on 9 out of 13 objects of the dataset.

### 5.5. TUD-L Dataset State of the Art Comparison

The TUD-L dataset contains three household objects under challenging light conditions. We summarize the bop performance score [63] (as we described in Section 5.2) in Table 4. Object 2 (Frog) does not have an outstanding contour difference in different poses, and thus its pose is harder to be estimated than the other two objects. Still, our method shows superior performance against the other methods.

### 5.6. Runtime Evaluation

The frame processing rate achieved by state of the art methods is summarised in Table 5. The methods are tested with different hardware (GTX 1080 or Titan X Pascal). We used an RTX 2080 Ti, which is considered about 30% faster than Titan X Pascal [66]. Taken the hardware difference into consideration, our CNN should be able to run about 65/(1+30%)=50 fps in Titan X. To summarize, we show that our CNN for pose estimation performs favorably against the state-of-the-art not only in terms of accuracy but also in terms of speed.

## 6. Conclusions

In this work, we proposed SynPo-Net, a novel CNN-based approach for 6DoF object pose estimation trained exclusively with RGB synthetic images reduced to single-channel images in pre-processing. We support the idea that neural network architectures need to be adjusted to the specific task of pose regression instead of relying on network layouts designed for classification. We address the domain adaptation problem by transforming synthetic and real images into a new domain with increased similarity. The results of an extensive experimental evaluation support our claims. In an ablation study, we showed how each proposed change to the network increases the pose accuracy. The comparison on the LINEMOD and TUD-L datasets proves that our method outperforms the existing state of the art in both accuracy and inference time. In future work, we plan to use our CNN as the backbone network for multi-object pose estimation.

## Figures and Tables

**Table 1:** We evaluated the proposed contributions in an ablation study. The networks have been tested with the Driller object of the LINEMOD dataset using the Average Distinguishable Distance (ADD) metric with a threshold of 10%. Dynamic augmentation means that the random augmentations will be applied after the images have been loaded for training (as we also mentioned in the end of Section 4.2). The details of other modifications in the table are described in Section 4.1.1.

**Input resolution (448 vs. 224)**		*√*	*√*	*√*	*√*	*√*	*√*
**Replace Pooling layers**			*√*		*√*	*√*	*√*
**Lie algebra**				*√*	*√*	*√*	*√*
**Dynamic Augmentation**						*√*	*√*
**Other CNN structure adjustments**							*√*
**ADD 10**	14.06	15.57(+1.51)	18.01(+2.44)	19.95(+1.94)	22.22(+2.27)	41.75(+19.53)	53.7(+11.95)

**Table 2:** Evaluation results on the LINEMOD dataset using the ADD metric with a threshold of 10%, using RGB images only and no pose refinement. Higher is better. *: We trained YOLO6D [38] and Pix2Pose [39] using the same synthetic images as ours.

Training Data	Synthetic Images						Real Images				
Method	SSD6D [15]	Rambach [18]	Pix2Pose * [39]	YOLO6D * [38]	AAE [36]	OURS	Brachmann [65]	BB8 [16]	YOLO6D [38]	Posecnn [33]	Pix2Pose [39]
Ape	0.00	4.37	3.64	16.09	4.18	**23.14**	−	27.90	21.62	−	58.10
Benchvise	0.18	21.74	3.95	33.91	22.85	75.23	−	62.00	81.80	−	91.10
Cam	0.41	1.25	0.00	2.91	32.91	6.66	−	40.10	36.57	−	60.90
Can	1.35	2.09	16.55	20.98	37.03	65.05	−	48.10	68.80	−	84.40
Cat	0.51	2.54	20.18	27.14	18.68	36.22	−	45.20	41.82	−	65.00
Driller	2.58	12.46	28.96	24.66	24.81	53.70	−	58.60	63.51	−	76.30
Duck	0.00	4.78	0.23	20.17	5.86	19.54	−	32.80	27.23	−	43.80
Eggbox	8.90	1.43	0.00	2.31	81.00	3.86	−	40.00	69.58	−	96.80
Glue	0.00	7.38	7.29	15.00	46.17	41.80	−	27.00	80.02	−	79.40
Holepuncher	0.30	3.88	2.51	15.44	18.20	21.10	−	42.40	42.63	−	74.80
Iron	8.86	38.22	1.82	57.63	35.05	85.07	−	67.00	74.97	−	83.40
Lamp	8.20	27.35	30.15	26.75	61.15	78.65	−	39.90	71.11	−	82.00
Phone	0.18	5.39	31.94	14.80	36.27	63.61	−	35.20	47.74	−	45.00
Mean	2.42	10.22	11.32	21.43	32.63	44.13	32.30	43.60	55.95	62.7	72.40

**Table 3:** Results on LINEMOD dataset using the ADD metric with a threshold of 10%, when depth information is used for pose refinement. Higher is better.

Method		Ape	B.Vise	Cam	Can	Cat	Driller	Duck	E.Box	Glue	Holep.	Iron	Lamp	Phone	Mean
SSD6D [15]	+ P. ICP	65.00	80.00	78.00	86.00	70.00	73.00	**66.00**	**100.00**	**100.00**	49.00	78.00	73.00	79.00	79.00
AAE [36]	+ ICP	24.35	89.13	82.10	70.82	72.18	44.87	54.63	96.62	94.18	51.25	77.86	86.31	86.24	71.58
OURS	+ ICP	65.86	94.98	35.30	94.82	79.81	83.50	57.26	3.86	73.28	68.63	96.18	94.70	91.59	72.29

**Table 4:** Results on the TUD-L dataset using the bop performance score. Higher is better. (The result of AAE and Pixel2Pose are taken from bop website [63] on 31 July 2019).

Method	AAE [36]	Pixel2Pose [39]	OURS
Obj1 (Dragon)	−	−	47.35
Obj2 (Frog)	−	−	35.88
Obj3 (Watering Can)	−	−	59.80
mean	40.1	34.9	**47.67**

**Table 5:** Inference time of methods without refinement, according to Reference [36].

Method	fps
SSD6D [15]	12
AAE [36]	13 (RetinaNet)
	42 (SSD)
BB8 [16]	4
Brachmann [65]	2
YOLO6D [38]	50
OURS	**65**
